# Microbiome study of a coupled aquaponic system: unveiling the independency of bacterial communities and their beneficial influences among different compartments

**DOI:** 10.1038/s41598-023-47081-0

**Published:** 2023-11-11

**Authors:** Alberto Ruiz, Daniel Scicchitano, Giorgia Palladino, Enrico Nanetti, Marco Candela, Dolors Furones, Ignasi Sanahuja, Ricard Carbó, Enric Gisbert, Karl B. Andree

**Affiliations:** 1 Aquaculture Program, Institute for Research and Technology in Agroalimentaries (IRTA), Ctra. Poble Nou. Km 5.5, 43540 Ràpita, Spain; 2 Unit of Microbiome Science and Biotechnology, Department of Pharmacy and Biotechnology, University of Bologna, Via Belmeloro 6, 40126 Bologna, Italy; 3 Fano Marine Center, The Inter-Institute Center for Research on Marine Biodiversity, Resources and Biotechnologies, Viale Adriatico 1/N, 61032 Fano, Pesaro Urbino Italy

**Keywords:** Microbiology, Ocean sciences

## Abstract

To understand the microbiome composition and interplay among bacterial communities in different compartments of a coupled freshwater aquaponics system growing flathead grey mullet (*Mugil cephalus*) and lettuces (*Lactuca sativa*), 16S rRNA gene amplicon sequencing of the V3–V4 region was analysed from each compartment (fish intestine, water from the sedimentation tank, bioballs from the biological filter, water and biofilm from the hydroponic unit, and lettuce roots). The bacterial communities of each sample group showed a stable diversity during all the trial, except for the fish gut microbiota, which displayed lower alpha diversity values. Regarding beta diversity, the structure of bacterial communities belonging to the biofilm adhering to the hydroponic tank walls, bioballs, and lettuce roots resembled each other (weighted and unweighted UniFrac distances), while bacteria from water samples also clustered together. However, both of the above-mentioned bacterial communities did not resemble those of fish gut. We found a low or almost null number of shared Amplicon Sequence Variants (ASVs) among sampled groups which indicated that each compartment worked as an independent microbiome. Regarding fish health and food safety, the microbiome profile did not reveal neither fish pathogens nor bacterial species potentially pathogenic for food health, highlighting the safety of this sustainable food production system.

## Introduction

The future of inland aquaculture requires that sustainability and efficiency rank as primary concerns. Aquaponic systems integrate the production of edible plants and animal proteins in a system that utilizes minimal water, which can make this system attractive for developing inland fish farms^1^. Among farmed fish species, the flathead grey mullet (*Mugil cephalus*) has a particular appeal in terms of sustainability, since it is a low trophic species, and its dietary protein requirement is lower when compared to the strictly carnivorous Mediterranean species that are currently farmed^2^. *M. cephalus* has been identified as a suitable species for feeding human populations in developing countries, even though it is also highly appreciated in developed countries due to its potential to produce both flesh and valuable processed by-products (salted mullet roe, also known as ‘bottarga’)^3^ with high nutritional values^4,5^. According to FAO aquaculture statistics^6^, a total of 11,938.6 tn of *M. cephalus* were produced in 2021, with Indonesia (46.3%), Israel (16.8%) and China (11.8%) being the main producers of this euryhaline species in extensive and semi-intensive aquaculture conditions^3^. A recent study from Rossi et al.^7^ evaluating the suitability of different marine fish species for multitrophic aquaponic systems using a multicriteria analysis, revealed that gilthead seabream (*Sparus aurata*), European seabass (*Dicentrarchus labrax*) and *M. cephalus* were the most appropriate species for such farming systems. Furthermore, the former authors stated that *M. cephalus* represents an interesting alternative to *S. aurata* and *D. labrax* from the perspective to promote sustainable aquaculture diversification due to its low trophic position. However, no practical information exists about the performance of this species under aquaponic farming technology.

Aquaponics systems can be either coupled in a single closed loop configuration, or decoupled in which the plant growth units are separated from the fish culture units^8^. All aquaponics systems have a modular design that includes the tanks for housing fish and tanks for growing plants; sedimentation and sludge removal systems and a biofiltration module. Either type of aquaponic system has an advantage over recirculation aquaculture systems (RAS) in that the plants utilize for their growth the excess of nitrogen compounds derived from the waste of the fish tanks; thereby, maintaining an improved water quality and optimizing the use of water resources for fish and plant production^9^.

Since RAS and aquaponic systems are engineered for biological output, their success depends in part on operationally controlling microbial activities^10^. Bacterial communities are not only intrinsic to but also fundamental for the proper functioning of both RAS and aquaponic systems^11^. Several reviews on the microbiome composition of RAS units have been published, which were mainly focused on the biofiltration process^10,12,13^. Coupled or decoupled aquaponic units are more complex culture systems than RAS, potentially having more diverse microbial communities and, consequently, the microbiome in the various compartments is likely to differ^9^. Several studies have shown that the microbial community in each aquaponic compartment (i.e., fish tank, biofilter, sump, hydroponic table, plant roots among others) is quite particular, with specialized groups of bacteria linked to different microbial functions in each specific niche sample. The composition of bacterial communities in different aquaponic’s compartments vary, which can be influenced by environmental conditions *(*i.e., pH, alkalinity, temperature), plant variety, fish stocking density, and nutrient abundance^11,14–16^. According to Kushwaha^16^, there is a need for a more thorough characterization of microbial communities linked with biochemical and system parameters to fully understand their physiological role in aquaponic systems. Among them, the bioconversion of nitrogenous compounds is of the largest importance to aquaponics systems^11^. There is limited understanding on the mechanisms of ammonia removal in zero-exchange aquaculture systems like coupled aquaponics. In these systems the removal of ammonia–nitrogen may be either photoautotrophic, autotrophic bacterial or heterotrophic bacterial-based, or a mixture of the former three^10^. Optimizing water quality and for effectively managing an aquaculture system, it is important to understand what type of ammonia removal occurs in the system. For this reason, providing insight into the microbial communities and their functions within aquaponic systems is of interest. In this context, two phylogenetically distinct groups of bacteria are collectively involved in the nitrification process. These two groups of bacteria are generally described as chemosynthetic autotrophic bacteria because they derive their energy from inorganic compounds, whereas heterotrophic bacteria derive energy from organic compounds^17,18^. In particular, ammonia oxidizing bacteria obtain their energy by catabolizing un-ionized ammonia to nitrite and include bacteria of the genera *Nitrosomonas*, *Nitrosococcus*, *Nitrosospira*, *Nitrosolobus*, and *Nitrosovibrio*, whereas nitrite oxidizing bacteria oxidize nitrite to nitrate, and include bacteria of the genera *Nitrobacter*, *Nitrococcus*, *Nitrospira*, and *Nitrospina*^17,18^. The major factors affecting the rate of nitrification are the ammonia and nitrite levels, the carbon/nitrogen ratio, the dissolved oxygen, pH, temperature, and alkalinity values. Furthermore, the overall conditions in an aquaponic unit need to consider the profile of these microbial communities as they have direct effects on the performance and health of the plant and animal species cultured within, as well as on food safety issues^19^. The introduction of distinct fish and plant species in a particular environment will bring with them different microbial communities as well, meaning that every combination of plant and fish in an aquaponics system can be unique. Thus, different arrangements of plants and aquatic animals can stimulate the growth of a wide variety of bacterial communities making predictions of the profile of the communities an unknown^20^. What is known is that plants release through root tissues a wide array of compounds, including organic acids, phenolics, nucleotides, carbohydrates, putrescine, sterols, and vitamins^21,22^. Root exudates, passively or actively released by plants as a stress response, protection or signalling, have the potential to influence the systems microbiome and the cultured fish, as similar phytochemicals have been found to affect bacterial abundance and diversity and fish health and behaviour^21^, which can variously act as both chemical attractants and repellents for microorganisms and remodel the microbiome composition in the aquaponic system in unexpected ways. In their turn, the microorganisms can metabolize fish wastes and uneaten feed liberating nutrients for uptake by plant roots^23^. Thus, plants and microorganisms communicate with each other just as the gut-brain axis can be used by intestinal microbiota to signal their host^24^.

However, as Kushwaha et al.^16^ recently reviewed, studies characterizing the microbial communities in aquaponics systems are limited. More outcomes on the microbiota from the different niches/compartments of aquaponic systems are needed to improve their control and homoeostatic balance. Whitin this scope, the knowledge of the diversity and functional distribution of these microorganisms in aquaponics systems has a key to understanding the functioning of these integrated farming systems and to enhance their performance. Furthermore, today the microbial community of the intestine of an animal can be modified for the benefit of the host by the introduction of probiotic organisms or prebiotic compounds into the feed^25^. In a similar manner, aquaponic units might be improved if the correct proportions of specific functional groups of bacteria were introduced to the system^21^, just as a biofilter system gets primed by seeding with nitrogen metabolizing bacteria^26,27^. By the introduction of specialized cohorts of bacteria, not only plant growth might be boosted, but also fish health and its performance can be improved. To move in the direction of an improved understanding of the microbiome of aquaponics systems, we have set-up a freshwater aquaponics system utilizing *M. cephalus* and lettuces (*Lactuca sativa*) seedlings to document how the microbiome in such a system develops. Under this scenario, we hypothesized that due to the water continuum within the different functional compartments composing a coupled aquaponic system, microbial composition among different compartments may be similar regardless of the complexity of the system even though species-specific differences may occur for each compartment within the aquaponic unit.

The objective of this study was to evaluate the farming of *M. cephalus* and *L. sativa* in an aquaponic coupled system to improve understanding of the microbial diversity of each compartment of such a system and demonstrate the productivity and safety of aquaculture farming practices using a sustainable low-trophic fish species like the flathead grey mullet during the winter season. The main reason for this choice was that most of the aquaponic studies published to date have been conducted under optimal farming conditions for both fish and plants^16^, whereas there is limited information about the performance of these aquaculture systems and their microbial communities when water temperature conditions are suboptimal for the fish but supportive for plant growth.

## Results

### Fish and plant growth

Results of flathead grey mullet growth are shown in Table 1. As expected, due to the low water temperature regimens at which the trial was conducted, fish grew poorly during the 99 days trial (*P* > 0.05). However, fish showed no outward signs of poor health, and their survival was 100% for all replicates. Regarding lettuce growth, all varieties grew very well within the hydroponic unit as yield results indicated in Table 1. In particular, lettuce ranged from 96.7 to 100.0% within the three aquaponic units. No signs of nutrient deficiencies (i.e., browning, chlorosis, discolouration among others) were observed in the leaves of the three varieties of lettuce grown; there was no need to supplement water in the S4 with minerals.

**Table 1:** Survival, growth performance in body weight (BW) and standard length (SL), and specific growth rate (SGR) of flathead grey mullet (*Mugil cephalus*) and lettuce (*Lactuca sativa*) reared in freshwater aquaponic system during winter conditions.

	Aquaponic unit 1	Aquaponic unit 2	Aquaponic unit 3
Fish			
Initial BW (g)	44.1 ± 10.3	43.5 ± 12.9	44.0 ± 12.0
Initial SL (cm)	13.1 ± 1.1	11.0 ± 1.2	11.0 ± 1.2
Final BW (g)	49.7 ± 11.4	48.6 ± 12.0	49.5 ± 14.4
Final SL (cm)	13.8 ± 1.0	12.0 ± 2.4	14.4 ± 1.2
SGR (% BW/day)	0.114	0.105	0.111
Survival (%)	100	100	100
Lettuce			
Romaine yield (g/lettuce)	345 ± 113	384 ± 110	386 ± 97
Iceberg yield (g/lettuce)	321 ± 74	335 ± 110	310 ± 126
Red leaf yield (g/lettuce)	182 ± 70	154 ± 69	192 ± 85
APY (kg/m^2^)	3.6	4.4	3.9
Survival (%)	98.9	100	96.7

*APY* aquaponic plant yield.

### Alpha and beta diversity of microbiome samples

**Figure 1:**
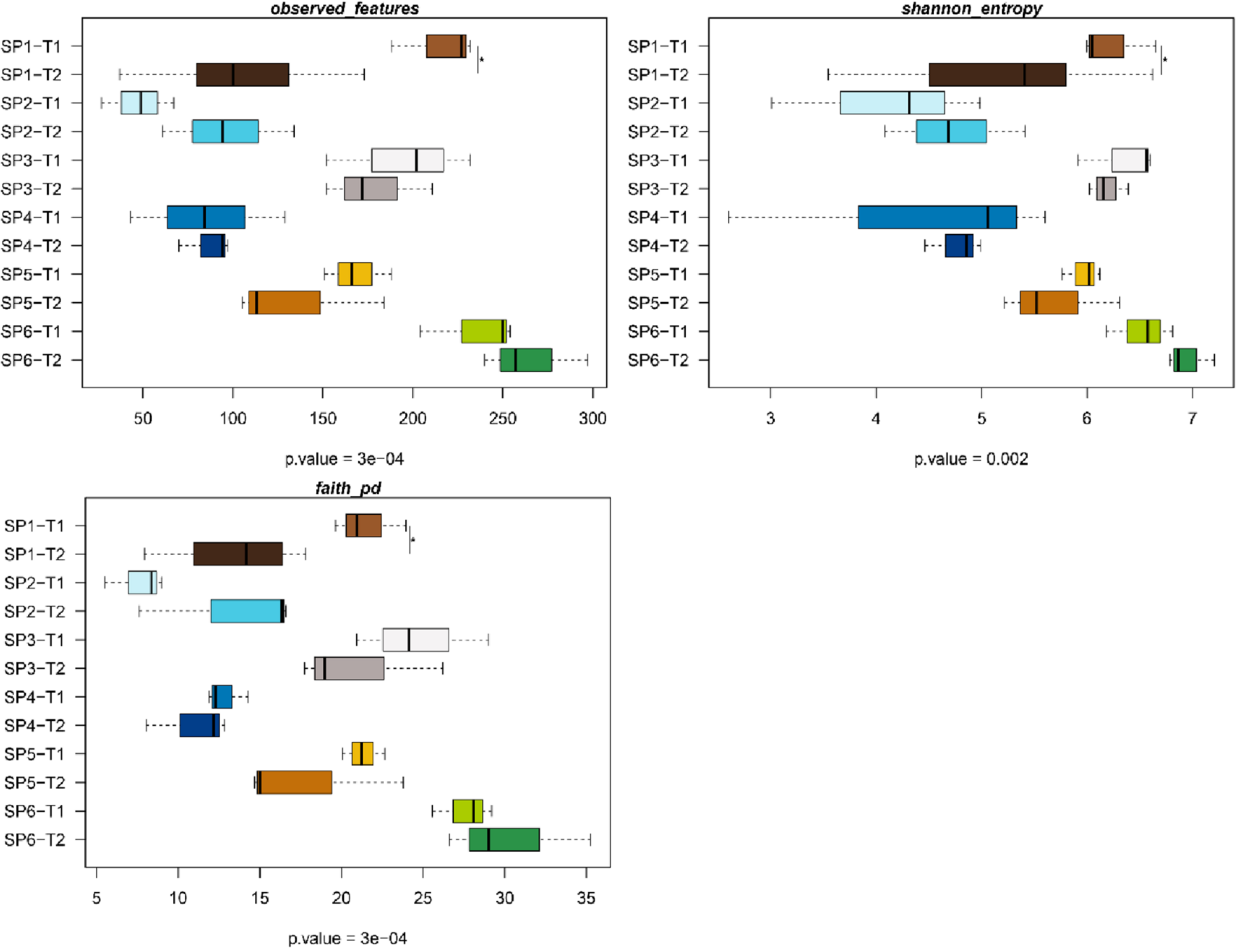
Boxplots representing the different used indices for alpha diversity of samples of all compartments from the aquaponics unit analysed. Sampling: SP1, flathead grey mullet intestinal content; SP2, water from the solid sedimentation tank; SP3, bioballs from the biological filter tank; SP4, water from the hydroponic unit; SP5, lettuce roots; SP6, biofilm from walls of hydroponic unit; T1, initial sampling point (2 weeks after fish acclimation); and T2, final sampling point (99 days).

After isolating DNA from each compartment using the best choice of DNA isolation kits, which are optimized for each specific matrix considered in our study (i.e., fish gut microbiome, water, biofilms and plant roots), we conducted 16S rRNA gene amplicon sequencing of the V3–V4 region**.** The alpha diversity indices (observed features, Shannon entropy and Faith’s phylogenetic diversity) of samples from different aquaponics’ compartments did not change between both sampling points, except for the fish intestines (SP1), whose diversity decreased at T2 (P < 0.05), while increasing in dispersion values with respect to T1 (Fig. 1). A similar trend, though not significant due to sample dispersion, was observed for the alpha diversity of microbiota measured in lettuce roots (SP5) (P > 0.05). When comparing among all types of samples, the biofilm from the walls of the hydroponic tank (SP6) and the microbiota associated to the bioballs from the biofilter (SP3) had the highest diversity in terms of richness (observed features), diversity in term of species richness and evenness (Shannon), and phylogenetic diversity (Faith diversity index) (P < 0.05); whereas the water from the solid sedimentation tanks (SP2) and from the hydroponic unit (SP4) were the ones with the lower diversity for the above-mentioned indices (*P* < 0.05). The alpha diversity of the lettuce (SP5) at both sampling times was closer to that of the water from both compartments, overlapping also with the values for the fish intestines from the end of the trial (T2). On the other hand, the intestinal microbiota of flathead grey mullets (SP1) at the initial sampling point (T1) was more similar to that of the bioballs (SP3) and of the biofilm from the hydroponic tanks (SP6).

**Figure 2:**
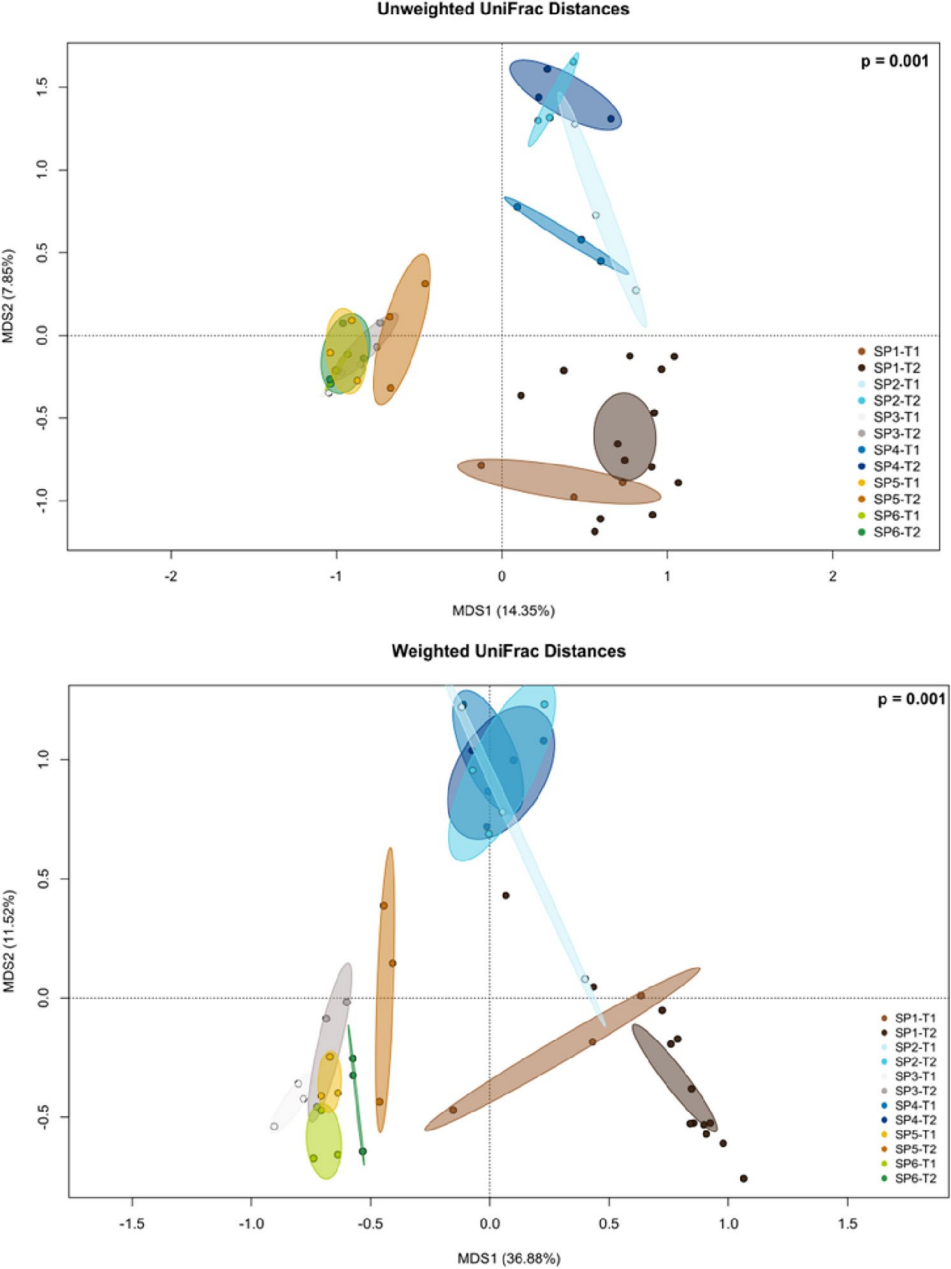
MDS analysis based on unweighted and weighted UniFrac distances of compartmental samples from the aquaponic units. Colours of ellipsoids correspond to the sample type and time of sample collection. Sampling: SP1, flathead grey mullet intestinal content; SP2, water from the solid sedimentation tank; SP3, bioballs from the biological filter tank; SP4, water from the hydroponic unit; SP5, lettuce roots; SP6, biofilm from walls of hydroponic unit; T1, initial sampling point (2 weeks after fish acclimation); and T2, final sampling point (99 days).

Regarding microbial structure, when qualitatively considering the phylogenetic relationships among ASVs between samples (unweighted UniFrac distances), water samples from both the sedimentation tank (SP2) and the hydroponic unit (SP4) markedly changed when T1 and T2 were compared (Fig. 2). Samples from flathead grey mullet intestinal contents (SP1) and lettuce roots (SP5) also displayed a substantial change during this 3-month trial, while those of bioballs (SP3) and biofilm (SP6) were hardly altered, with both types of samples resembling each other regardless the sampling point. When quantitatively assessing the phylogenetic relationships among ASVs, also considering the abundance of each ASV (weighted UniFrac distance), similar results were obtained for beta diversity dissimilarities of the fish intestinal contents (SP1) and lettuce (SP5) between the T1 and T2 sampling points, although a larger dispersion among samples was found. However, the biofilm from the walls of the hydroponic unit (SP6) and the biofilter balls (SP3) showed lesser overlapping areas when comparing T1 and T2 with this index. Furthermore, in this case the water from both sampled compartments (SP2 and SP4) showed a lower change over time than when using unweighted UniFrac distances, and the microbial structure of both water samples resembled each other regardless the sampling time (SP2 *vs.* SP4). On the other hand, irrespective of the index used for approaching beta diversity from the samples’ bacterial communities, there may be a trend where the free-living pelagic taxa cluster together (water samples) apart from the benthic/epiphytic taxa found attached to substrates such as the bioballs, wall biofilm and plant roots, with the fish gut samples forming a third cluster, and this structure was maintained at the first and final sampling times (Fig. 2).

**Figure 3:**
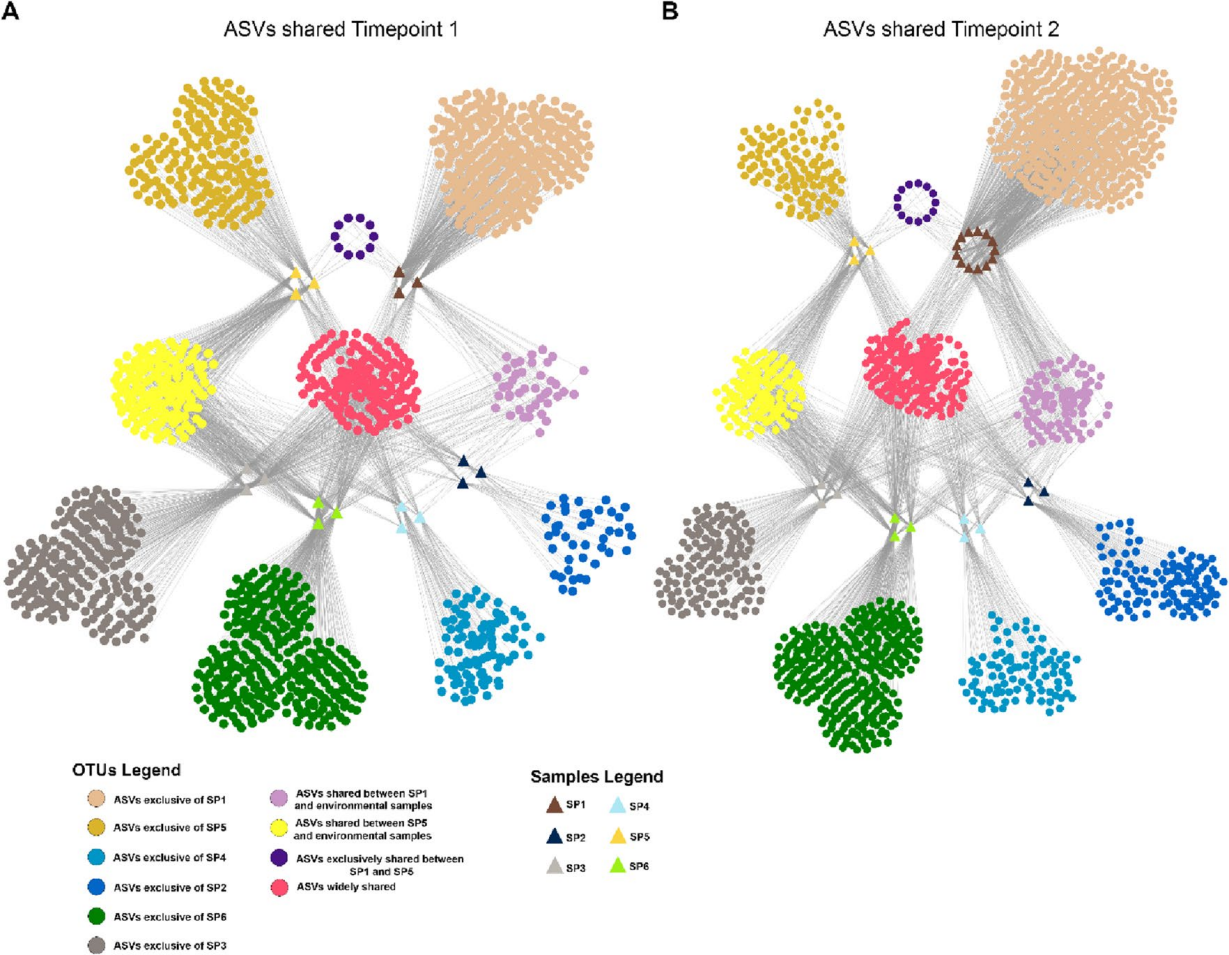
Networks of samples within the dataset at both T1 (**A**) and T2 (**B**) based on ASVs clustered at 97% similarity. Dot and triangle size is fixed, while colour legend representing shared or exclusive ASVs is reported in the bottom part of the figure. Sampling: SP1, flathead grey mullet intestinal content; SP2, water from the solid sedimentation tank; SP3, bioballs from the biological filter tank; SP4, water from the hydroponic unit; SP5, lettuce roots; SP6, biofilm from walls of hydroponic unit. T1, initial sampling point (2 weeks after fish acclimation); and T2, final sampling point (99 days).

Even if this different segregation regarding the different ecosystems has been shown, at ASVs level, clustered at 97% similarity, we identified several ASVs shared between host-associated microbiomes (fish gut and roots) and environmental microbiomes (water, bioballs, and biofilm samples) at both T1 and T2 (Fig. 3). Specifically, there has been an increase in ASVs exclusively shared among fish gut and environmental samples (no identification in root samples), from T1 (42 ASVs) to T2 (89 ASVs) Fig. 5. While a reduction in terms of the number of ASVs exclusively shared among root and environmental samples (no identification in fish gut samples) has been observed moving from T1 (167 ASVs) to T2 (135 ASVs). At the same time, an increase in the ASVs exclusively shared between fish gut and root samples has been observed moving from T1 (10 ASVs) to T2 (13 ASVs), as well as an increase in ASVs widely shared among almost all samples, always from T1 (157 ASVs) to T2 (174 ASVs).

### Composition of bacterial communities

**Figure 4:**
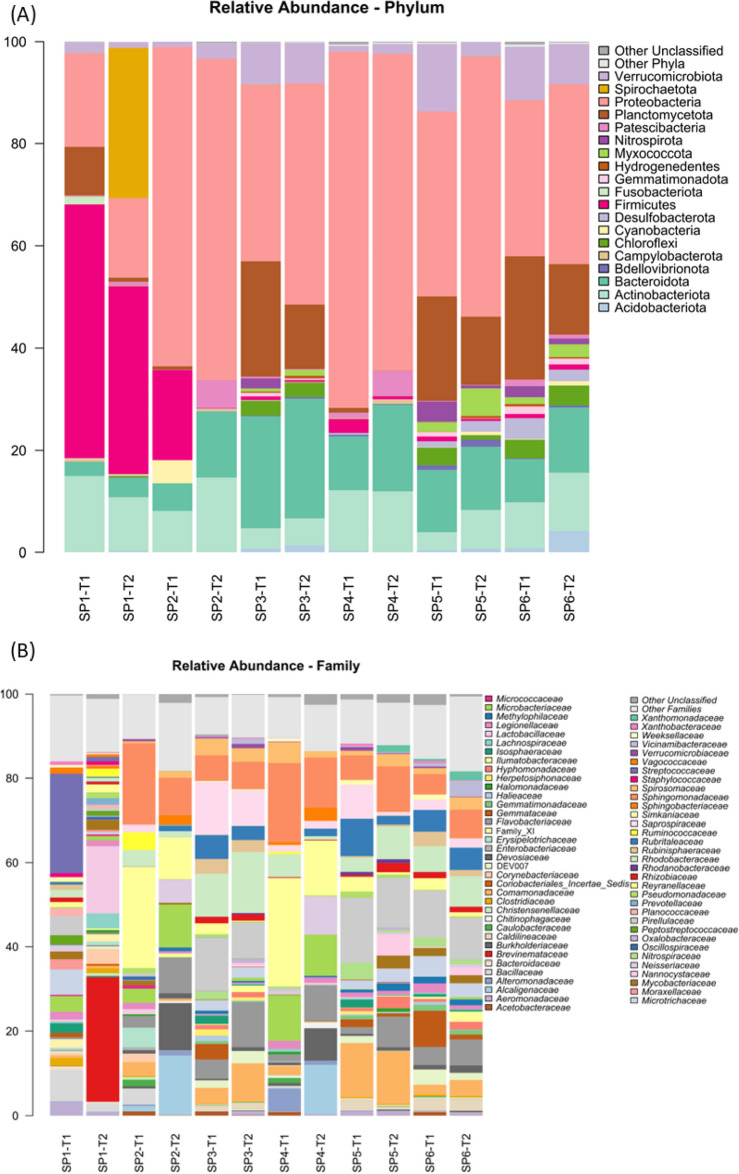
Relative abundance of bacteria at phylum (**A**) and family (**B**) levels from the samples from the aquaponic units at the initial (T1) and final (T2, 99 days) sampling points. Taxa with an abundance < 0.5% in at least 2 samples are classified as Other Phyla while those with an unknown classification at phylum level are classified as Other Unclassified. Taxa appearance in the figures are in alphabetical order from bottom to top. Sampling: SP1, flathead grey mullet intestinal content; SP2, water from the solid sedimentation tank; SP3, bioballs from the biological filter tank; SP4, water from the hydroponic unit; SP5, lettuce roots; SP6, biofilm from walls of hydroponic unit.

Regarding the relative abundance at the level of phylum (Fig. 4, Supplementary Table 1), four main phyla from fish intestinal contents (SP1) were found at T1, these being Firmicutes (49.7%), Proteobacteria (18.3%), Actinobacteriota (15.0%) and Planctomycetota (29.4%). The main phyla from bioballs (SP3), biofilm (SP6) and plant roots (SP5) from the hydroponic unit included Proteobacteria (34.5%, 30.5%, and 36.1% respectively), Planctomycetota (22.6%, 24.1% and 20.5%, respectively) and Bacteroidota (21.9%, 8.5%, and 12.2%, respectively). The Planctomycetota were nearly absent as a dominant group from the water samples (SP2 and SP4) and the T2 in the fish gut contents (SP1). Furthermore, Spirochaetota appeared as a dominant group (29.4%) in fish gut contents at the end of the trial (T2), whereas a decrease in Firmicutes (36.8%) in fish intestinal contents (SP1) and in the water of the sedimentation tank (0.09%) (SP2) with respect to T1 (17.6%) was also noticed.

**Figure 5:**
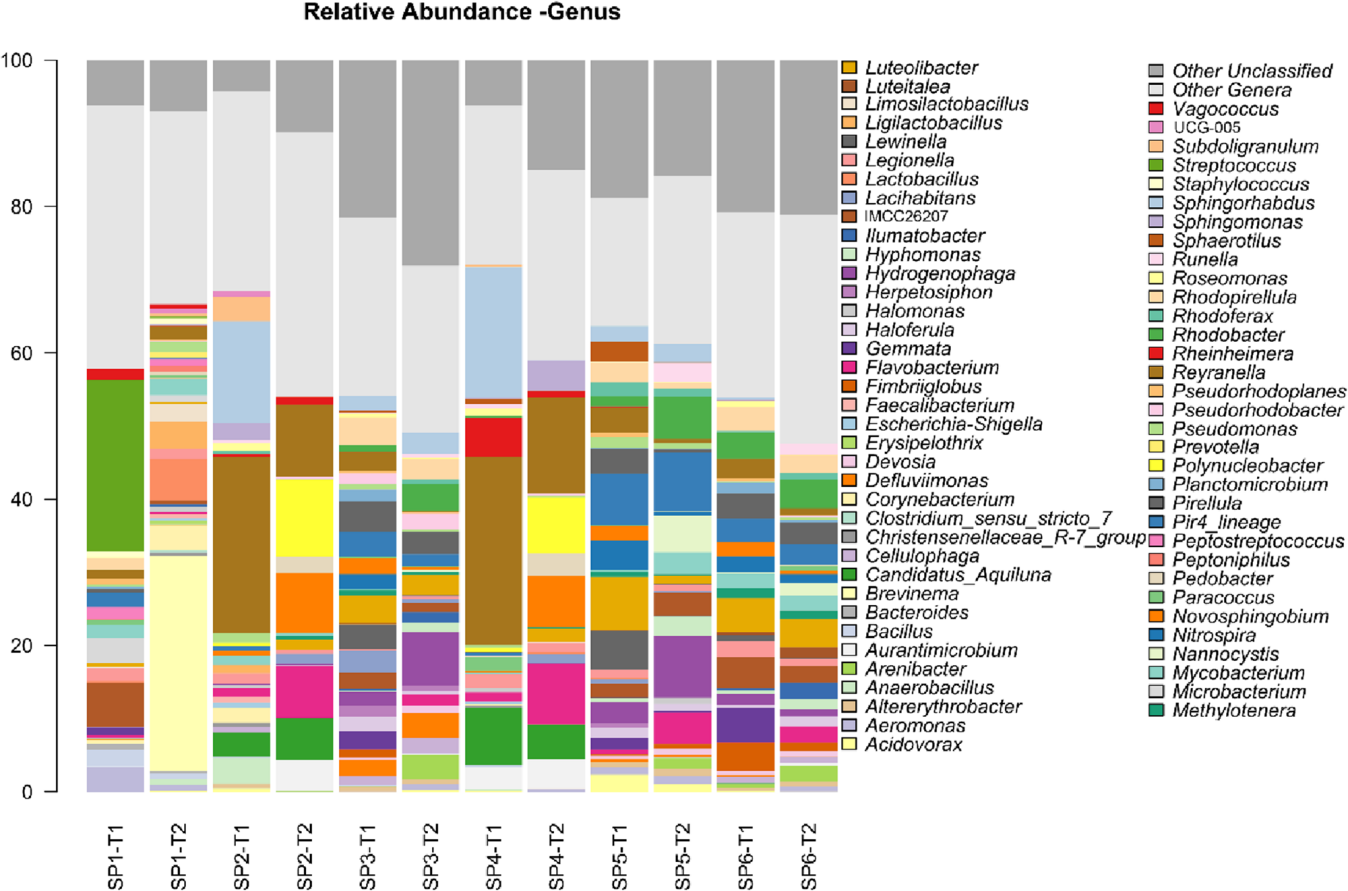
Relative abundance of bacteria at genus level from the samples from the aquaponic units at the initial (T1) and final (T2, 99 days) sampling points. Taxa with an abundance < 1% in at least 2 samples are classified as Other Genera while those with an unknown classification at genus level are classified as Other Unclassified. Taxa appearance in the figures is in alphabetical order from bottom to top. Sampling: SP1, flathead grey mullet intestinal content; SP2, water from the solid sedimentation tank; SP3, bioballs from the biological filter tank; SP4, water from the hydroponic unit; SP5, lettuce roots; SP6, biofilm from walls of hydroponic unit.

The relative abundance of bacterial families among samples (Fig. 4, Supplementary Table 2) showed an unusual abundance of the *Brevinematacea* in the fish intestines at T2 with respect to T1, an increase that ranged from 0 to 29.4%, which corresponded to the above-mentioned increase in Spirochaetota at a higher taxonomic level, and it also included *Brevinema* sp. at the genus level (Fig. 5, Supplementary Table 3). At T1, there was a dominant presence of *Streptococcaceae* in the fish intestines (SP1) that disappeared by T2 (from 23.6 to 1.1%) and was nearly absent from samples from the rest of analysed compartments. At T2, the relative abundance of the family *Lactobacillaceae* greatly increased (from 0.5 to 16.0%) in flathead grey mullet intestinal contents (SP1). Other statistically significant changes in relative abundance at family level were detected between T1 and T2 from fish gut samples (SP1) in terms of *Microtrichaceae* (6.0 to 0.5%), *Bacillaceae* (7.3 to 2.1%), *Isosphaeraceae* (2.3 to 0.0%), *Neisseriaceae* (1.4 to 0.0%), *Aeromonadaceae* (3.4 to 0.8%), and *Moraxellaceae* (2.3 to 0.5%) (Supplementary Fig. 1; P < 0.05). The family *Flavobacteriaceae* was a lesser dominant group among all the samples collected at T2, but it was nearly absent from fish gut samples (SP1) at both time points. The families *Burkholderiaceae* and *Alcaligenaceae* were dominants in the water samples from the sedimentation (SP2) and hydroponic (SP4) tanks at T2, but nearly absent in T1. Remarkable was also the common composition at the family level such as *Rubritaleaceae, Comamonadaceae, Sphingomonadaceae, Rhodobacteraceae, Rhizobiaceae, Acetobacteraceae, Caldilineaceae, Flavobacteriaceae, Spirosomaceae, Saprospiraceae* and *Microtrichaceae* of the samples from bioballs (SP3), lettuce roots (SP5) and biofilm growing on the walls of the hydroponic unit (SP6). Similarly, the water samples of both compartments (SP2 and SP4) also shared many families within a same range of relative abundance values among them in both sampling times (i.e., *Legionellaceae, Sphingomonadaceae, Rhodobacteraceae, Reyranellaceae, Flavobacteriaceae, Spirosomaceae*, and *Microbacteriaceae*).

Among dominant genera, the outstanding results among fish intestinal contents were the decreased relative abundance of *Streptococcus* sp. after T1 (from 23.5 to 0.3%), and the increase in *Brevinema* sp. at T2 (0.0 to 29.4%); moreover, they were the only genera exclusively found in fish gut. There were also statistically significant changes in abundance from fish gut samples for *Haloferula* sp. (0.09 to 0.01%), *Aeromonas* sp. (3.4 to 0.8%), *Pirellula* sp. (0.5 to 0.02%), and IMCC26207 (6.0 to 0.5%) from T1 and T2 sampling points (Supplementary Fig. 2; P > 0.05). At T1, *Sphingorhabdus* sp. was a dominant genus (17.9 and 13.9%, respectively) in both water samples (SP2 and SP4), but it was absent by T2 in both water samples. Furthermore, while *Reyranella* sp. was a dominant genus in T1 in SP2 and SP4 (25.7 and 24.1%, respectively), their abundance decreased by T2 (13.1 and 9.9%, respectively).

## Discussion

Grey mullet (family Mugilidae) farming has a tradition of several hundred years both in Oriental and Western countries in extensive and semi-intensive systems; however, there is still scarce information about their performance and nutritional requirements under different rearing systems and conditions^28^. This group of euryhaline and eurythermal species is generally farmed extensively and semi-intensively in earthen ponds or in cages^29,30^; nonetheless, there exists very limited information about its potential culture in other farming systems. Due to the low trophic level and adaptability to different rearing conditions, grey mullets are considered among the most suitable and sustainable species to be farmed in aquaponic systems^7^. Thus, this study attempted to rear *M. cephalus* in a coupled freshwater aquaponic system and evaluate the microbial composition of different compartments within the aquaponic to further understand the functioning of these complex systems during the winter season, under suboptimal fish thermal conditions.

Regardless of being an eurythermal species with a wide geographical distribution^31^, *M. cephalus* under current experimental conditions did not show any increase in somatic growth during the trial that lasted 99 days. These results may be attributed to the low water temperatures at which the trial was run (14.2 ± 3.3 °C) and the reduction in feeding behaviour and stress response associated to these low temperatures^32^, since the optimal temperature values for this species are comprised between 20 and 26 °C^23^. Fortunately, this study was not only focused on evaluating the performance *of M. cephalus* in aquaponic conditions during winter, since we were also interested in the output of lettuce production within the system under such thermal conditions. Thus, yield values of lettuce under current aquaponic conditions (3.6–4.4 kg/m^2^) compared favourably to those reported in open fields (4.6 kg/m^2^) and in soil-less rooftop urban home gardens (3.9 kg/m^2^) in the same climatic region, whereas they were slightly lower compared to mineral fertigation practices (5.4 kg/m^2^)^33,34^. The relevance of current results lies on the absence of the use of fertilizers for lettuce growth within the aquaponic unit that are produced because of fish metabolism and bacterial activities^26^. Apart from these promising vegetal production results, further research is needed to test these practices under warmer temperatures.

Regarding the microbiome composition of different compartments within the aquaponic system, bacterial communities are strongly determined by a wide array of biotic (i.e., plant variety, fish species, fish stocking density) and abiotic (i.e., water pH, alkalinity, temperature, nutrient levels) factors, and their proper characterization is essential for optimal functioning of aquaponics^14–16,35^. In this sense, after DNA isolation from different aquaponics’ compartments using the best choice of DNA isolation kits depending on the specific matrix considered in our study (i.e., fish gut microbiome, water, biofilms and plant roots) and conducting 16S rRNA gene amplicon sequencing of the V3–V4 region, it was not surprising that under the current experimental conditions the microbial communities from the gut samples were the most unstable over time in terms of alpha diversity. This was due to that the fact that they depend not only on environmental factors, such as the decreasing temperatures over the course of the trial, but also on the effect of the fish physiological adaptations to such environmental changes^36,37^. On the other hand, the unweighted and weighted UniFrac results showed that bacterial compositions in different compartments within the aquaponic unit were significantly different. In agreement to several studies^11,14,15,38^, differences found between the water samples, biofilm-bioballs, plant roots and fish intestinal content samples indicated that each sampled compartment worked with an independent microbiome due to the low, or almost null, number of shared ASVs among sampled groups as depicted in Fig. 5. Regarding fish, there is scarce information about the composition of flathead grey mullet gut microbiota. Bertini et al.^2^ found that Fusobacteria and Proteobacteria were the most abundant phyla in the intestine of flathead grey mullet accounting for 70% of the total variability in fish fed diets containing graded levels of bacterial single cell protein and reared in low salinity water (7‰)^2^. In contrast, the microbiome composition from migratory *M. cephalus* of the Northwest Pacific coast were dominated by Proteobacteria, Firmicutes and Actinobacteria^39^. These results are different from those obtained in the current study where Firmicutes (36.8%), Spirochaetota (29.4%) and Actinobacteriota (15.0%) appeared as dominant groups at T2. The above-mentioned differences between both studies may be related to abiotic factors like water temperature^36^ and salinity^40^, and biotic ones like the diet^2,41^, the genetic origin of fish^39^ as well as their age and/or sex^42^ among others. In the current study, although Firmicutes and Actinobacteriota abundances remained stable as the main dominant groups between T1 and T2 sampling points in flathead grey mullet intestinal samples, there was a decrease in Planctomycetota (∆ = − 8.6%) and a dramatic increase in Spirochaetota (family Brevinematacea; ∆ =  + 29.4%) abundances. The increase in Spirochaetota in intestinal contents was surprising when compared to the other compartments, considering that it was only found at very low densities in lettuce roots (0.02%) at T1. This phylum has been reported to be a minority in other omnivorous freshwater species^43, 44^, whereas spirochaetes have also been suggested to play an important ecological role in herbivorous fish^45^. However, *Brevinema* sp. has also been reported to be among the dominant taxa in the gut microbiota of the anadromous Artic char *Salvelinus alpinu*s^46^ and in the euryhaline tiger puffer *Takifugu rubripes*^47^. Among Spricohaetota, this group includes anaerobic and facultatively anaerobic spirochetes that are indigenous to various types of aquatic environments including bottom sediments of ponds and marshes in a free-living state; their existence does not depend on physical associations with other organisms^48^. The change in Brevinematacea (*Brevinema* sp.) was coupled with a decrease in Streptococcaceae (*Streptococcus* sp.) in fish intestines at the end of the trial (∆ = − 23.2%) that was nearly absent from samples from the rest of analysed compartments. Similar low *Streptococcus* sp. abundances have been found in the gut microbiome of flathead grey mullet^2^. Several strains of *Streptococcus* sp. are pathogenic to fish species^49,50^; thus, the decrease in relative abundance of these bacteria may be considered as beneficial for the host^51^, which might be attributed to an increase of probiotic bacteria like *Ligilactobacillus* sp. (∆ = + 3.7%) and *Lactobacillus* sp. (∆ = + 5.5%)^49,52^. Due to the shift in relative abundance in Brevinematacea (*Brevinema* sp.) and Streptococcaceae (*Streptococcus* sp.) from flathead grey mullet intestinal samples, and their almost absence in the other compartments of the aquaponic unit analysed, we hypothesize that the above-mentioned changes might be attributed to the presence in the water of metabolites exuded by lettuce roots that may modulate the growth of some bacteria in fish guts such as those belonging to the genera *Brevinema* and *Streptococcus* spp. as different studies have shown that these compounds have the capacity to modify the soil microbiota and their exudation is promoted by flooding among other factors^21,53^.

Regarding the microbiome composition of water samples, Proteobacteria, Actinobacteriota and Bacteroidota were the most abundant phyla in the water samples from the sedimentation tank and hydroponic unit at T1 and T2 sampling points. In particular, these phyla accounted for 92.2% and 90.1% of total bacterial variability at this taxonomic level at T1 and T2 in water samples from the sedimentation tank, respectively, whereas in water samples from the hydroponic unit they represented 76.1% and 90.4% of total bacterial variability. In this sense, there was an important reduction in the relative abundance of the representatives of the Firmicutes phylum in water samples from the hydroponic compartment between T1 and T2 (∆ =  − 17.5%), with *Bacillaceae, Erysipelotrichaceae, Lactobacillaceae* and *Ruminococcaceae* families being nearly absent at T2 (0.08%). Although bacterial communities were quite stable at the level of phylum in water samples from the sedimentation tank and the hydroponic unit between T1 and T2 regardless of changes in water temperature, dissolved oxygen levels and pH values (Supplementary Fig. 1) along the trial, there were several changes in different Proteobacteria families. For instance, there was an increase in relative abundance in *Alcaligenaceae* (∆ =  + 11.5%), *Neisseriaceae* (∆ =  + 8.4%) and *Burkholderiaceae* (∆ =  + 7.1%) coupled with a decrease in abundance in *Reyranellaceae* (∆ =  − 12.7%), *Sphingomonadaceae* (∆ =  − 6.8%) and *Rhodobacteraceae* (∆ =  − 4.6%). Furthermore, only *Flavobacteriaceae* increased in abundance (∆ =  + 6.8%) within the Bacteroidetes phylum. The increase in abundance of representatives from the *Alcaligenaceae* and *Burkholderiaceae* families may be associated to the amount of organic matter in the water in both compartments^54,55^; organic matter that may come from uneaten feed pellets and fish faeces and are used by heterotrophic bacteria as nutrient and energy sources^9, 10^. In addition, the increase in abundance of Neisseriaceae may be linked to an enhancement of the denitrification process taking place in water because of the presence of nitrates originated from nitrifying bacteria in the biological filter^56^.

When considering the microbial composition of the biological filter, Proteobacteria, Planctomycetota and Bacteroidota were the main phyla accounting for most of the microbial diversity in bioballs at T0 and T1 (79.0% and 79.4%, respectively). These groups of bacteria are commonly found in the biofilters of different RAS and aquaponics set-ups^10–15^. There are two groups of bacteria that collectively perform nitrification; these groups are generally categorized as chemosynthetic autotrophic bacteria because they derive their energy from inorganic compound, whereas the second group are heterotrophic bacteria that obtain their energy from organic compounds^18,19^. In particular, Proteobacteria display a high degree of metabolic diversity and are associated with nutrient cycling and the remineralization of organic matter, while assimilatory and dissimilatory nitrate reduction are generally carried out primarily by members of Planctomycetes, and Bacteroideota have been shown to be an important heterotrophic bacterial phylum involved in the cycling of complex carbon and protein-rich substances^10^. Although microbiota composition in terms of the main phyla from bioballs was really stable between both sampling points, there was a decrease in Planctomycetota (∆ =  − 9.9%) coupled with an increase in Proteobacteria (∆ =  + 8.8%) at T1. The two genera that showed the larger increase in relative abundance between T1 and T2 sampling points were aerobic denitrifying bacteria; in particular, the heterotrophic *Rhodobacter* sp. (∆ =  + 2.7%) and the autotrophic *Hydrogenophaga* sp. (∆ =  + 5.5%), whereas another denitrifying genera involved in nitrate reduction like *Pseudomonas* sp. remained stable. These aerobic autotrophic genera have been described as denitrifiers in the biological filters of RAS units and in zero-discharge biofloc-based recirculating aquaculture systems^57^. Furthermore, changes in heterotrophic bacteria abundance like *Rhodobacter* sp. may be explained by different abiotic factors, since the major factors that affect the rate of nitrification (pH, alkalinity, temperature, oxygen, ammonia, and salinity), also play a dominant role in heterotrophic bacterial growth^18,19^. Regarding nitrifying bacteria, *Nitrosomonas* sp. abundance increased from 0.35 to 1.5% (4 times; ∆ =  + 1.15%) between T1 and T2, even though their relative abundance values were lower in comparison to other groups of bacteria, whereas other nitrifying bacteria remained stable (i.e., DSSD61, oc32 and MND1). Furthermore, *Nitrospira* spp., which is involved in the oxidation of nitrite to nitrate^17^, slightly decreased (∆ =  − 2.0%) and *Hydrogenophaga* spp., a genus implicated in denitrification^58^ increased by T2 (∆ =  + 5.5%). Some of these genera are typical from biological filtration systems in aquaculture units^57^, and their change in abundance may be associated to the maturation of the biological filtration unit and changes in abiotic factors^10,12,13,18,19^, as well as to their interaction and/or competition with heterotrophic microorganisms^11,17^. The biofilm growing in the walls of the hydroponic unit was mainly composed of Planctomycetota, Proteobacteria, Bacteroidota and Actinobacteriota that represented 72.2% and 73.1% of the bacterial diversity in terms of phyla at T1 and T2, respectively. Similar results were reported in biofilm samples from the fish tank of a semi-closed loop aquaponic system growing Nile tilapia (*Oreochromis niloticus*) and plants^14^. When comparing bacterial diversity among both sampling points, there was a significant decrease in abundance of Planctomycetota families like *Xanthomonadaceae, Sphingomonadaceae, Comamonadaceae, Hyphomonadaceae* and *Rhodobacteraceae* (∆ =  − 10.4%) coupled by an increase in *Vicinamibacteraceae* (Acidobacteriota; ∆ =  + 2.9%) as well as the other dominant phyla (∆ =  + 2.5–4.7%). *Vicinamibacteraceae* are a group of biofilm-forming bacteria that has been found in soil, water, and sediments in oligotrophic and eutrophic conditions^59–61^; thus, their presence in our samples would be correlated to their chemoorganoheterotroph metabolism and the presence of organic compounds derived from feed and fish faeces in the water. In addition, the increase in abundance of several representatives of Planctomycetota in biofilm samples may be attributed to their metabolic versatility and the autotrophic metabolic capacities of some genera^62,63^.

The bacterial community growing in lettuce roots was the second most diverse in terms of ASVs as shown in Fig. 3, being dominated at the phylum level by Bacteroidota, Planctomycetota, Proteobacteria and Verrucomicrobiota (82.1% and 79.5% of total bacterial diversity at T1 and T2, respectively). Similar results in terms of the dominant phyla were reported in different aquaponic trials when describing the microbiota composition of lettuces in a coupled aquaponic system with different tilapia species^9,11,14^, even though slight differences in their relative abundance were found among studies. The above-mentioned phyla, particularly Proteobacteria, Actinobacteria, and Firmicutes have been identified as plant growth promoting bacteria^64^. These results were different from those described by Day et al.^38^ where Proteobacteria was the dominant phylum (> 95%) in lettuce roots from an aquaponic unit that was inoculated with a commercial bacterial inoculum containing nitrifying bacteria. At the genus level, the most abundant at T2 were bacteria from the *Hydrogenophaga* genus (8.4%), followed by representatives from the *Pirellula*-related Pir4 clade (7.9%), *Rhodobacter* spp. (5.8%) and *Flavobacterium* spp. (4.4%) (Flavobacteriaceae). *Hydrogenophaga* sp. has been reported in the roots of lettuce grown in aquaponic systems^11,15^ or irrigated with eutrophic^65^ and wastewater^66^, as well as in the biological filter^57^. The family of *Comamonadaceae* is known for its chemoorganotrophic or chemolithoautotrophic nutrition, using H_2_ as energy source and CO_2_ as a carbon source, which has been described as a plant growth promotor, essentially by acting in the nitrogen cycle (denitrification and N_2_ fixation)^67^. Furthermore, the *Pirellula*-related Pir4 lineage (family *Pirellulaceae*) was the second genus in abundance in lettuce roots and this is the first study that reports its presence in an aquaponic system. This group of freshwater aquatic bacteria inhabits low oxygen habitats^68^, although it has also been found in the soil and roots of different crops^69^. Regardless of the strong aeration present in the hydroponic unit within our aquaponic system (61–70% oxygen saturation), the presence of *Pirellula*-related Pir4 bacteria is suggestive of the complexity and conformation of the roots and rhizosphere where different groups of bacteria may inhabit different microbial microhabitats^70^. Regarding *Rhodobacter* spp. (*Rhodobacteraceae*), bacteria from this genus have been found in the water of the fish rearing tank^14^ and in the biofilter^9^ of the aquaponic system and RAS units^57^, but never reported in relevant relative abundances in plant roots in aquaponic units. The presence of these bacteria may be attributed to their chemoheterotroph metabolism and their involvement in the mineralization of wastes within the system. The noted increase of *Rhodobacter* spp. in lettuce roots seen in this study may also be related to quorum sensing, or rather a breakdown of such intercellular communication. The production of homoserine lactone auto-inducers by *Rhodobacter* spp. has been documented previously^71^, but there has also been noted the activity of mycorrhizal fungi that degrade these quorum-sensing compounds in terrestrial settings^72^. Given the absence of soil after transfer to the aquaponics unit, the eventual decrease in terrestrial mycorrhizal fungi might lead to a resumption of intercellular communication among the *Rhodobacter* cohort thereby augmenting their growth as seen at T2 relative to T1 (∆ =  + 4.4%). Considering *Flavobacterium* sp. (*Flavobacteriaceae*), this genus is associated with the capacity to degrade complex organic compounds and mineralize solid waste, thereby providing soluble micronutrients for the plants which may be beneficial for the plant in terms of health and growth^11^. *Flavobacterium* sp. represents an important part of the root-associated microbiome in a broad range of plant species, and it has been also reported in the lettuce root microbial community in aquaponics^9,11,14^. Finally, when considering changes in the relative abundance of bacteria in lettuce roots during the trial, we found remarkable changes in Proteobacteria (∆ =  + 14.8%) and Verrucomicrobiota (∆ =  − 10.5%) and Planctomycetota (∆ =  − 7.1%). Those changes were characterized by an increase in relative abundance of the representatives of *Nannocystis* (∆ =  + 5.0%)*, Rhodobacter* (∆ =  + 4.4%)*, Flavobacterium* (∆ =  + 3.7%)*, Mycobacterium* (∆ =  + 2.8%) and *Runella* (∆ =  + 2.6%) spp. coupled with a decrease in *Lewinella* (∆ = -5.3%), *Nitrospira* (∆ = − 3.4%), *Reyranella* (∆ =  − 2.9%) and *Pirellula* (∆ =  − 2.9%) spp. Those changes in microbial composition may be attributed to several factors like the inherent evolution in the nutrient content within the hydroponic unit along the grow out process^11^, the competition for resources among bacteria with similar metabolism^11,15^, and lettuce growth and root exudates that play a key role in microbial development in the root zone^21,22,73^. Furthermore, changes in abiotic water parameters may be also responsible for the above-mentioned changes in bacterial abundance; in particular, dissolved oxygen, nitrate and sulphate have a strong influence in the aerobic compartments of aquaponic units^9^.

In addition to considering how different microbiomes proliferate and crosstalk within the aquaponic system, it is also relevant to evaluate where this system may represent a risk in terms of food health and food safety. In this context, numerous microbes and coliforms that naturally degrade organic compounds and fish waste in the biofilters of aquaponic systems have been documented; however, the presence of many bacteria that may cause diseases in fishes, plants, and humans is also significant^19^. Plant or fish pathogenic bacteria can significantly impact hydroponic production techniques and yields. Therefore, food safety assessment in production systems is becoming increasingly vital in management and harvesting activities. For instance, bacteria such as *E. coli* and *Salmonella* spp. have been identified as risk assessment indicators for water quality and products in aquaponics^16^.

The information about bacterial pathogens of *Mugil cephalus* is scarce, in recent episodes of mass mortalities in Egypt^74^ *Photobacterium damselae* subsp*. piscicidae* was incriminated as the putative agent; however, as in most cases, there was not a clear aetiological agent, but rather a polymicrobial and multifactorial etiology. Likewise, Burke and Rodgers^75^ had associated the red-spot disease in flathead grey mullet with both *Vibrio anguillarum* and *V. alginolyticus*, and with *Aeromonas hydrophila* as a secondary pathogen. Under current conditions, neither of these halophytic genera (*Photobacterium* and *Vibrio* spp.) were detected in the gut of flathead grey mullet. Moreover, *Aeromonas* sp. decreased 2.56% in the gut between the two sampling times, with a negligible 0.84% abundance at T2. These results are encouraging, since they indicate that the commonly found pathogens for this species might have difficulty in proliferating in this farming system, if in the fish are evidenced as passive carriers, or to enter in a freshwater environment. *Enterobacter cloacae* has been associated with both human health problems and mortalities in this species^76^; in our system, the *Enterobacter* genus was not a matter of concern, since it contributed with a minimal 0.09% to the fish gut microbiome at T1 and was not detectable at the end of the assay. Regarding *Lactococcus* sp., a modest increase in abundance of 0.67% was seen in the fish gut between T1 (0.11%) and T2 (0.78%). Interpreting this is challenging at the genus level, since *Lactococcus* species can be pathogenic (i.e., *L. garvieae*) for mugilid species^77^ causing great mortalities, while other *Lactococcus spp.* are regarded as beneficial for their probiotic capacities^78^; in any case their ASV abundance values were low under current farming conditions. Another potential concern for fish health, *Pseudomonas* sp., was not seen restricted to the fish gut compartment, on the contrary it was found across the system always at very low abundances. Therefore, regarding the distribution profile and abundance of the genera which could represent pathogenic species, our aquaponic setup seemed to provide a healthy environment. This would also be the case regarding food safety indicators, such as *Escherichia-Shigella*, which were scattered in the system at very low relative abundance levels, below regulatory levels^69^. Moreover, neither *Listeria* sp. nor *Salmonella* sp. were found in any aquaponics’ compartment, results that agreed with Fox et al.^19^ when evaluating real bacterial counts in a demonstrative aquaponic system. Matching the fish gut compartment results, none of the bacterial indicators for food safety (*E. coli, Salmonella* spp. and *Listeria* spp.) have been found in the lettuce roots, backing recent work regarding the safety of aquaponic products, even indicating that no food poisoning outbreaks had been attributable to aquaponic’s system^9, 19^.

## Conclusions

Understanding the biological processes that shape the structure and dynamics of microbiomes during the maturation of an aquaponic system is a key element for optimizing the functioning of these complex farming systems as well as providing insight into the interaction of microbial populations. The bacterial composition of a freshwater coupled aquaponic unit farming flathead grey mullet and lettuces differed among different aquaponic compartments (fish intestine, water from the sedimentation tank, biological filter, water and biofilm from the hydroponic unit, and lettuce roots), with independent microbiomes associated to each compartment that showed a low, or almost null, number of shared ASVs among sampled groups, suggesting that each compartment creates a specific environmental niche. However, this study also showed that the diversity of the benthic and epiphytic bacteria that adhered to the biofilm from the bioballs within the biological filter, hydroponic tank walls, and lettuce roots resembled each other, while that of free-living pelagic bacteria from the water samples also clustered together; both of the groups were separated from that of the gut bacterial communities. Moreover, this study shed light on the modulation of the composition of the bacterial community among different compartments. For instance, the increase of nitrifying bacteria in the biofilms from the biofilter balls and the hydroponic tank walls was notable and this potentially counteracts the fish ammonium production (Planctomycetota members); and further, the denitrifying bacteria that contributed to the biofilter maturation (*Rhodobacter* sp. and *Hydrogenophaga* sp.) or that were present in the water (Neisseriaceae) probably were in response to nitrates released by the nitrifying bacteria from the biofilter. In a similar manner, the organic matter released by fish residues (faeces and/or uneaten feed) may have modified the microbiota of the water from the sedimentation and hydroponic tanks (promoting Burkholderiaceae and Alcaligenaceae members), and of the biofilms adhered to the walls of the lettuce tanks (promoting growth of Vicinamibacteraceae members) and to their roots (indicated by Rhodobacteraceae members). These results in turn hinted at a possible modulation of the bacterial populations by root exudates from the plant roots and fish guts. Furthermore, the presence of *Hydrogenophaga* sp. and *Flavobacterium* sp. in the roots was a good indicator of the successful growth performance of the lettuce planted during the trial. The extremely low, or near absence, of ASVs corresponding yo risks by  pathogens or food safety, is a very interesting aspect for this new food production system. Future studies using proteomics and/or metabolomics should give rise to a better understanding of the mechanistic processes underlying the relations and relative abundances of the various microbial taxa in each aquaponics compartment.

## Methods

### Aquaponics unit design and farming conditions

**Figure 6:**
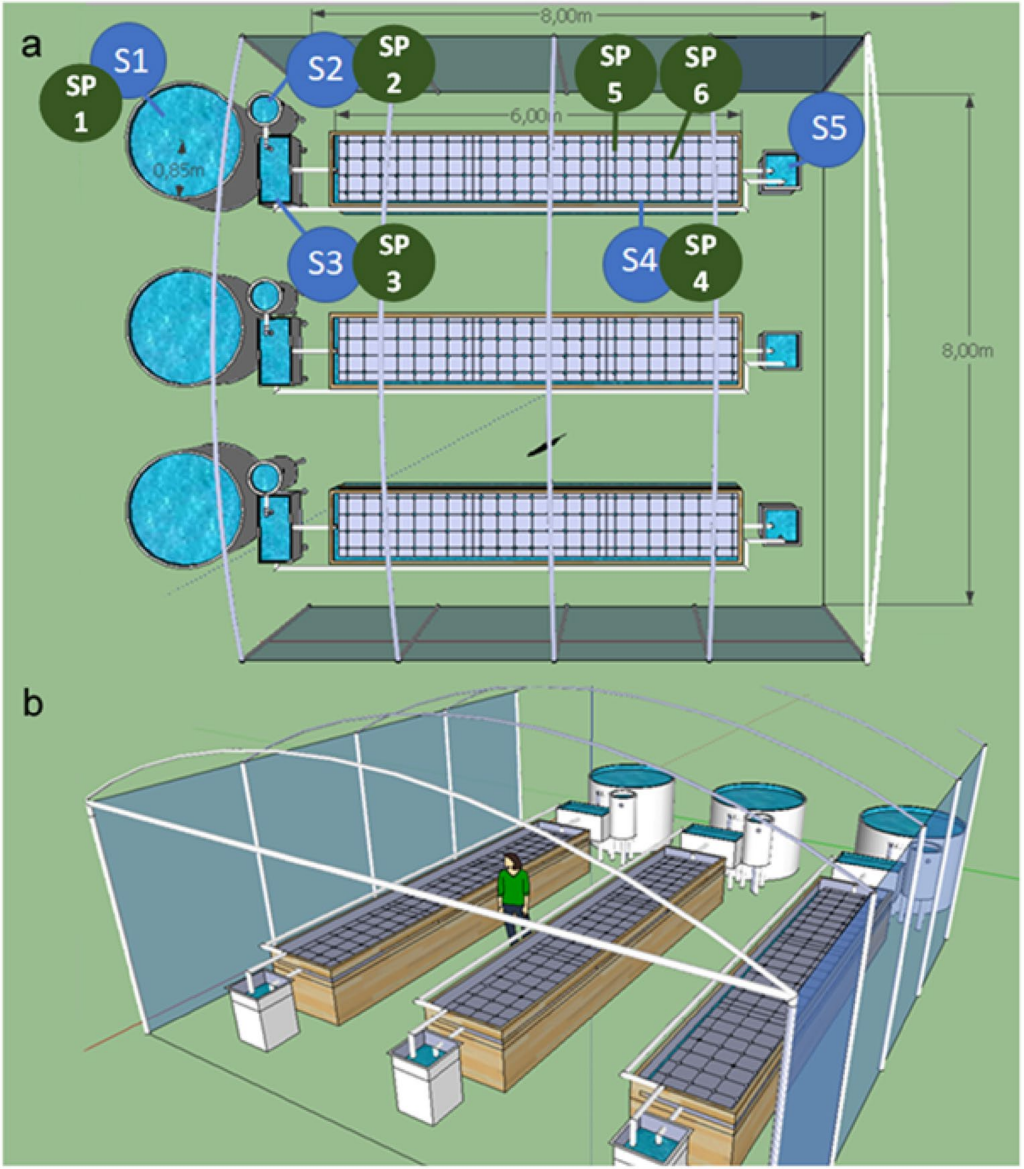
Illustration of the experimental set-up for the aquaponic units and the points where samples were collected from each compartment for DNA extraction and microbiome analysis. Compartments of the aquaponic system: S1, fish rearing tank; S2, solid sedimentation tank; S3, biological filter; S4, hydroponic unit; and S5, water pump. Sampling: SP1, flathead grey mullet intestinal content; SP2, water from the solid sedimentation tank; SP3, bioballs from the biological filter tank; SP4, water from the hydroponic unit; SP5, lettuce roots; SP6, biofilm from walls of hydroponic unit.

Figure 6 depicts the schematic representation of the experimental system used in the current study, which is composed of three replicate aquaponic units within a greenhouse. Each aquaponic unit is comprised of a 2000 L cylindroconical tank where fish are held (S1); followed by a sedimentation tank were suspended particles (uneaten pellets and fish faeces) are settled and removed (S2). Then, underground fresh water passes to a biological filter tank (125 L) full of bioballs where ammonia is converted into nitrites and nitrates by the action of nitrifying bacteria (S3). After this, water flows by gravity to the hydroponic unit (S4) (1.2 × 6.0 × 0.45 m, width × length × height; surface = 7.2 m^2^; functional water volume = 2.9 m^3^) where plants are supported in polystyrene rafts covering the whole surface of the hydroponic unit. At this level, strong aeration is provided by an air blower (0.3 Kw h) to provide enough oxygen for the growth of plant roots. Finally, water is returned from the hydroponic unit to the fish rearing tank with a water pump (2,470 L/h; 0.1 Kw h) (S5). The whole volume of each aquaponic unit was 5 m^3^ (water turnover time for an entire circulation cycle: 2 h). No thermal control was provided neither for the aquaponic water or the greenhouse air, thus water and air temperature were ambient. This was an intentional choice to reduce running and maintenance costs, as well as promoting the transference of this farming technology to rural or underdeveloped areas.

In the hydroponic unit, the floating raft was stocked with lettuce plants from three different commercial varieties (romaine, red leaf, and iceberg) that were purchased at a local lettuce nursery (stage of three-four leaves) and planted in each aquaponic unit (n = 92 lettuces per aquaponic system; n = 30–31 lettuces from each variety). At their arrival, lettuce’s roots were washed with freshwater in order to remove the soil attached to them and prevent the introduction of soil particles into the system. Romaine lettuces were planted proximal to the biological filter tank, the middle third was stocked with red leaf lettuces and the distal section of the hydroponic unit was stocked with iceberg lettuces. No plant fertilizer was added into the system (foliar application or dissolved in water) since no signs of mineral deficiency (i.e., browning, chlorosis, dark green colour, discolouration, leaf deformation or die off among others) were detected from lettuce planting to harvesting at the end of the assay. Regarding fish, wild flathead grey mullet fry (24.2 ± 0.8 mm in standard length, SL; 202 ± 5 mg in wet body weight, BW; n = 3500) were obtained from Pescados y Mariscos Roset SL (Deltebre, Spain). Fish were pre-grown at IRTA research facilities until needed for different research purposes. For this trial, 342 fish were used (44.1 ± 10.1 g in BW, 13.1 ± 1.1 cm in SL). Fish were evenly distributed among the three fish rearing tanks within each aquaponic unit (n = 114 per tank; initial stocking biomass = 2.5 kg/m^3^) and acclimated within the system for 2 weeks. During this period, fish were fed with a commercial diet with 54% crude protein, 18% crude fat, and 19.5 MJ/kg digestible energy (T-Nutra 1.1 MP; 1.1 mm pellet size; Skretting, Spain). As water temperature progressively decreased during the trial (Supplementary Fig. 3), feeding ration was progressively reduced from 2.5 to 0.5% of the stocked biomass to reduce the amount of uneaten feed due to reduced feed intake. The trial started on 20/10/2020 and lasted 99 days including the 2 weeks-acclimation period of the fish (27/01/2021). This study was reported in accordance with ARRIVE guidelines^79^.

Water quality parameters (temperature, oxygen, and pH) were measured daily, whereas nitrogenous compounds in water (ammonia, nitrites, and nitrates) were measured twice a week. Water temperature and dissolved oxygen were measured with an OXI330, Crison Instruments (Spain), pH was registered with a pH meter 507, Crison Instruments, and ammonia, nitrite and nitrate levels were measured with a HACH DR 900 Colorimeter, Hach Company (Spain). Air temperature was measured by a mercury thermometer. Under current experimental conditions, average air temperature was 15.4 ± 7.7 °C (Supplementary Fig. 3) and followed the normal pattern for the season of the year when the trial was conducted (November to January). Regardless of the typical seasonal air temperature changes, water temperature was quite stable (14.2 ± 3.3 °C) as shown in the Supplementary Fig. 3. The rest of water quality parameters (ammonia, nitrites and nitrates) are shown in the Supplementary Fig. 4. In brief, water quality parameters during the trial were as follows: oxygen levels: > 90% saturation, salinity: 2.0 ± 0.2‰ and pH 7.1–7.5. Ammonia, nitrite, and nitrates values were 0.6 ± 0.05 mg/L NH_4_^+^, 2.0 ± 0.03 mg/L NO_2_^−^ and 25.0 ± 5.1 mg/L NO_3_^−^, respectively.

### Sample collection

At the end of the trial (day 99), all fish in each tank were anesthetized with MS-222 (100 mg/L) and BW were individually measured. Survival was determined by counting the number of fish at the end of the trial in relation to the initial number stocked in each tank at the beginning of the trial. The following equations were used for calculating common KPIs associated to fish and plant growth:

$${\text{Specific growth rate in body weight }}\left( {{\text{SGR}}, \, \% {\text{ BW}}/{\text{day}}} \right) \, = { 1}00 \, \times \, \left[ {\left( {{\text{ln BW}}_{{\text{f}}} {-}{\text{ ln BW}}_{{\text{i}}} } \right)/{\text{days}}} \right].$$

Aquaponics plant yield (APY, kg/m^2^) was calculated taking into consideration the yield of the aerial part of lettuces (in kg) per m^2^ in each aquaponic unit and the duration of the trial.

For the evaluation of the microbiota in the different aquaponics’ compartments, six sampling points (SP1–SP6) were established for collection of microbiome material from each aquaponic unit (Fig. 6). Samples were collected after fish acclimation to the system for two weeks (T1: 04/11/20) and at the end of the trial (T2: 27/01/21). For fish gut microbiota (SP1), a total of three and five fish per tank were sampled at the beginning and at the end of the trial, respectively. For this purpose, fish were netted from the tank (S1), euthanized with an overdose of anaesthetic MS-222 (300 mg/L) and eviscerated. Then, the full intestine (from the pyloric valve to the anus) was aseptically removed and opened lengthwise with sterile scissors and then the contents, including the mucosal layer, removed by gentle scraping using a round laboratory spatula. Similarly, water samples (3 replicates per aquaponic unit) were taken from S2 (SP2) and S4 (SP4) compartments at T1 and T2 times. In brief, water (50 mL) was filtered through 0.22 µm pore size-sterile filters (cellulose acetate membrane filters, 25 mm diameter; ref. 10404106; Millipore, MERK, Spain), then placed into 5 mL Eppendorf tubes and stored at − 80 °C. From the biofiltration compartment (S3), several bioballs (n = 3–4) (SP3) were collected and cut into small fragments using sterile scissors and stored in 5 mL Eppendorf tubes. Furthermore, root samples from romaine lettuces were collected from S4 (*ca*. 3 cm of the root’s distal part from red leaf lettuce) (SP5), and the biofilm (SP6) growing in the walls of the hydroponic unit (~ 20 cm below the waterline). The SP6 sample was collected with a sterile cotton swab, and its head was cut off using sterile scissors then placed into a 5 mL Eppendorf tube. All samples were stored in − 80 °C until further DNA extraction.

### Bacterial community DNA extraction, sequencing and bioinformatic analysis

For microbiota analyses, the DNA from each sample was extracted with different commercial kits. From the fish gut content, the DNA was extracted as described in Bertini et al.^2^; in brief, 300 mg of sample were weighted and extracted with a modified protocol of the DNeasy Blood & Tissue Kit (QIAGEN, Hilden, Germany). Total DNA from water filters was extracted using the DNeasy Power Water kit (QIAGEN, Hilden, Germany), total DNA from bioballs and swab biofilm samples from the tank wall was extracted following the DNeasy PowerBiofilm kit (QIAGEN, Hilden, Germany), while root samples were processed for DNA extraction following the DNeasy PowerBiofilm kit (QIAGEN, Hilten, Germany). Total DNA extracted from all samples was then quantified with NanoDrop ND-1000 (NanoDrop Technologied, Wilmington, DE) and stored at − 20 °C until further processing. DNA libraries were prepared following the Illumina “16S Metagenomic Sequencing Library Preparation” as recommended by the manufacturer (Illumina, San Diego, USA)^80^, amplifying the V3-V4 hypervariable regions of the 16S rRNA bacterial gene using the 341F (5ʹ-WTTACCGCGGCTGCTGG-3ʹ) and 785R (5ʹ-GACTACHVGGGTATCTAATCC-3ʹ) primers carrying Illumina overhang sequencing adapters and 2xKAPA HiFi HotStart ReadyMix (KAPA Biosystems). Libraries were normalized to 4 nM and pooled. Pooled libraries were denatured with 0.2 N NaOH and diluted to 6 pM with 20% PhiX control. Sequencing was performed on Illumina MiSeq platform using 2 × 250 bp paired-end protocol according to the manufacturer's instructions (Illumina, San Diego, CA). At the end of the sequencing process, raw sequences were processed using a pipeline combining PANDAseq and QIIME2. High-quality reads, obtained after a filtering step for length and quality with default parameters, were cleaned and clustered into amplicon sequence variants (ASVs) using DADA2. Taxonomy was assigned using a hybrid method combining VSEARCH and q2 classifier trained on the SILVA database release 138.1. Three different metrics were used to evaluate internal ecosystem diversity (alpha-diversity) − number of observed ASVs (observed features), Shannon entropy index, and Faith’s Phylogenetic Diversity (faith_pd). Unweighted and weighted UniFrac distances were computed to estimate inter-sample ecosystem diversity (beta-diversity) and used as input for Principal Coordinates Analysis (PCoA). ASVs were then clustered at 97% similarity to reconstruct ASV networks, which were then created on the data provided by the *make_otu_network* script provided by the QIIME pipeline. The representation was obtained using Cytoscape software (http://www.cytoscape.org/) and using Compound Spring Embedder (CoSE) as the layout for the ASVs.

### Statistical analysis

Changes in flathead grey mullet BW between the onset and the end of the trial were compared by means of a t-test (P < 0.05). Microbiota analysis and respective plots were produced using R software (https://www.r-project.org/) with “vegan” (http://www.cran.r-project.org/package-vegan/), “MADE4” and “stats” packages (https://stat.ethz.ch/R-manual/R-devel/library/stats/html/00Index.html). Data separation was tested by a permutation test with pseudo-F ratios (function “adonis” in “vegan” package). When required, Wilcoxon and Kruskal–Wallis test were used to assess significant differences in alpha diversity and taxon relative abundance between groups. When necessary, p-values were corrected for multiple testing with Benjamin–Hochberg method, with a false discovery rate (FDR) ≤ 0.05 considered as statistically significant.

### Ethics statement

All procedures involving fish and plant manipulation and tissue sampling from experimental animals complied with the Spanish (law 1078 32/2007 and Royal Decree 1201/2015) and ongoing European legislation (EU2010/63). The experimental protocol was authorized by the Ethical Committee of the Institute of Agrifood Research and Technology and the Generalitat of Catalunya, Direcció General de Polítiques Ambientals i Medi Natural (CEEA 11264/2021).

### Supplementary Information

Supplementary Figure 1.

Supplementary Figure 2.

Supplementary Figure 3.

Supplementary Figure 4.

Supplementary Table S1.

Supplementary Table S2.

Supplementary Table S3.

## Data Availability

Data availability on request to the corresponding author.
