# Supplementary figures and images for: Microbiome study of a coupled aquaponic system: unveiling the independency of bacterial communities and their beneficial influences among different compartments
Source: Sci Rep. 2023 Nov 11;13:19704. doi: 10.1038/s41598-023-47081-0 (PMC10640640; doi:10.1038/s41598-023-47081-0)

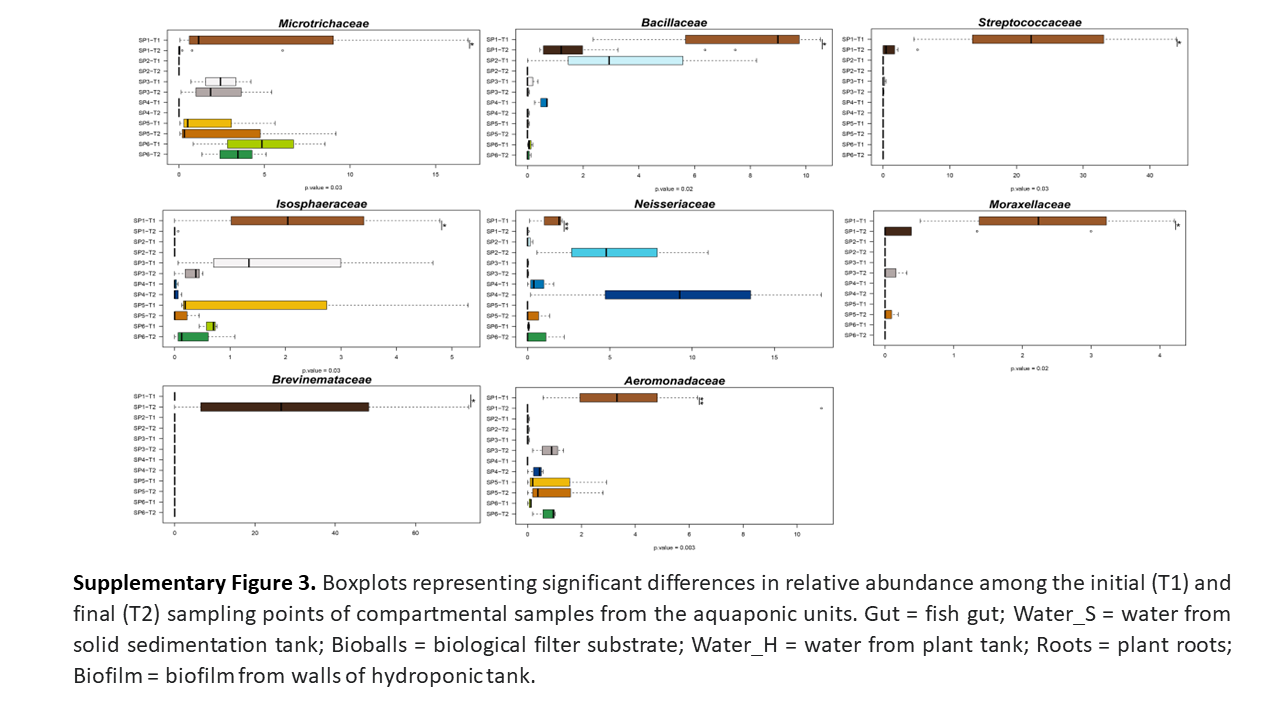

Supplement: Supplementary file 1 — Supplementary Figure 1. [file 41598_2023_47081_MOESM1_ESM.tif]

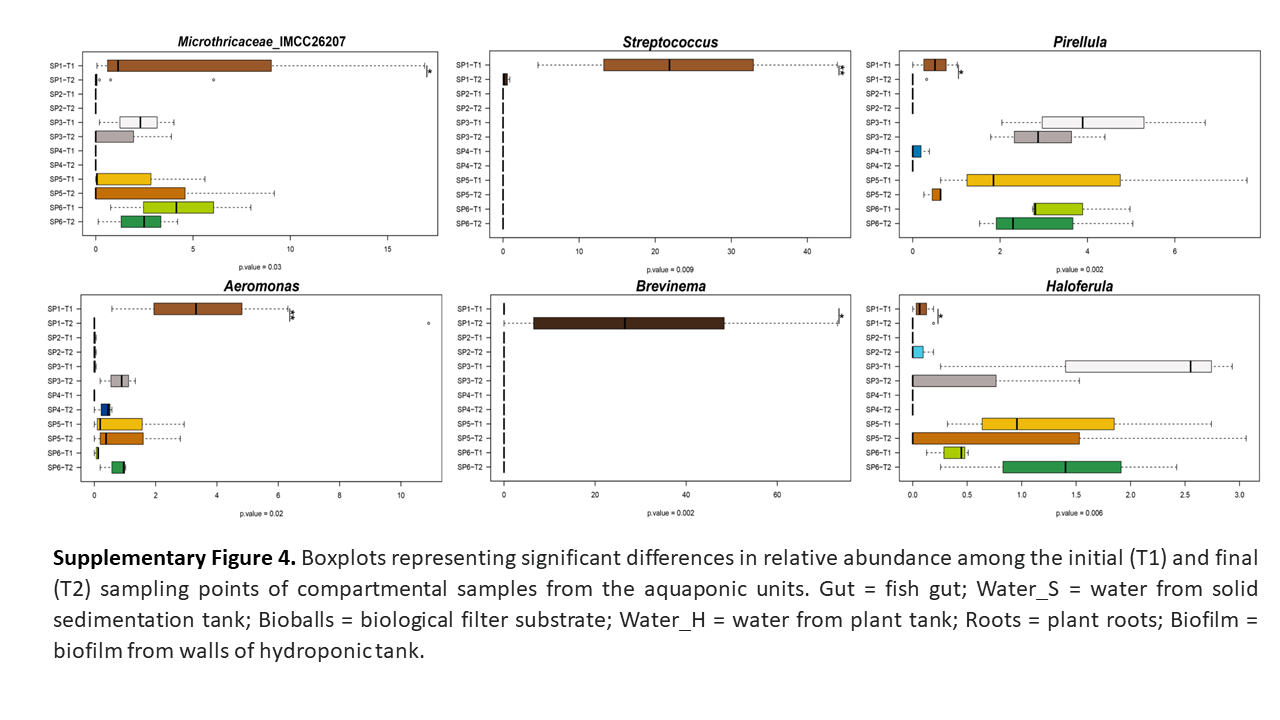

Supplement: Supplementary file 2 — Supplementary Figure 2. [file 41598_2023_47081_MOESM2_ESM.tif]

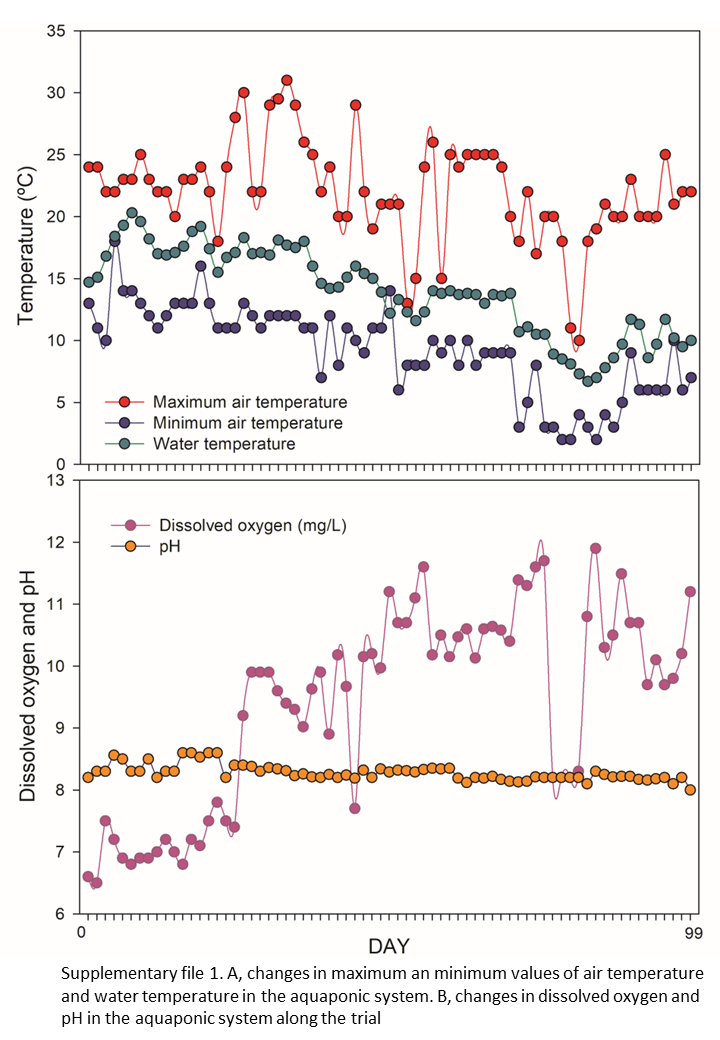

Supplement: Supplementary file 3 — Supplementary Figure 3. [file 41598_2023_47081_MOESM3_ESM.tif]

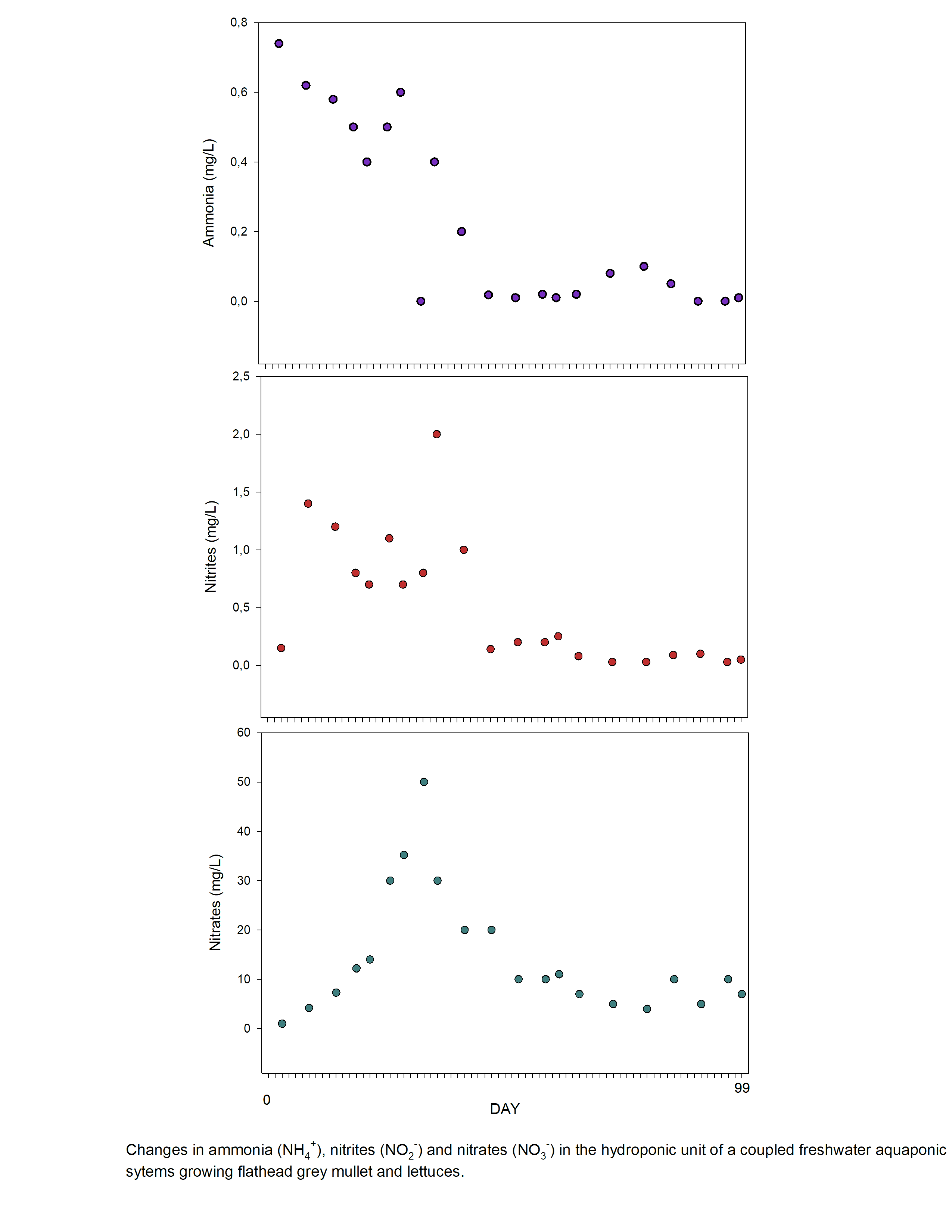

Supplement: Supplementary file 4 — Supplementary Figure 4. [file 41598_2023_47081_MOESM4_ESM.tif]
